# GPCRs in CAR‐T Cell Immunotherapy: Expanding the Target Landscape and Enhancing Therapeutic Efficacy

**DOI:** 10.1002/advs.202517188

**Published:** 2025-12-23

**Authors:** Zhuoqun Liu, Yuchen Xiao, Yamin Zhao, Lihe Dai, David J.H. Shih, Shikang Liang, Honglei Tian, Liu Liu, Feng Tian, Bing Liu, Lei Chang, Chao Zhang

**Affiliations:** ^1^ Fundamental Research Center Shanghai Yangzhi Rehabilitation Hospital (Shanghai Sunshine Rehabilitation Center) School of Medicine Tongji University Shanghai 201619 P. R. China; ^2^ Department of Gynecology The First Affiliated Hospital of Zhengzhou University Zhengzhou 450000 P. R. China; ^3^ Department of Urology Eastern Hepatobiliary Surgery Hospital Shanghai 201805 P. R. China; ^4^ School of Biomedical Sciences Li Ka Shing Faculty of Medicine The University of Hong Kong Pokfulam Hong Kong SAR 999077 P. R. China; ^5^ Division of Life Science The Hong Kong University of Science and Technology Clear Water Bay Hong Kong SAR 999077 P. R. China; ^6^ Shanghai Yuhui Pharmaceutical Technology (Group) Co., Ltd Shanghai 201203 P. R. China; ^7^ Hebei Key Laboratory of Medical Data Science Institute of Biomedical Informatics School of Medicine Hebei University of Engineering Handan 056038 P. R. China; ^8^ Department of Orthopedics and Precision Research Center for Refractory Diseases Shanghai Jiao Tong University Pioneer Research Institute for Molecular and Cell Therapies, Shanghai General Hospital Shanghai Jiao Tong University School of Medicine Shanghai 200080 P. R. China

**Keywords:** cancer, CAR‐T therapy, (GPCRs, antibody), immunotherapy

## Abstract

Chimeric Antigen Receptor T cell (CAR‐T) therapy has shown significant efficacy in treating hematologic malignancies. However, its application in solid tumors is still challenged by a scarcity of specific targets and the immunosuppressive tumor microenvironment. G protein‐coupled receptors (GPCRs), due to their wide distribution and diverse signaling cascades in tumorigenesis, have emerged as promising targets for CAR‐T therapy. This review systematically integrates recent advances of GPCR CAR‐T therapy for cancer immunotherapy, with a particular emphasis on current targeting strategies and optimization approaches. This includes the identification of GPCRs as novel tumor‐associated antigens to expand CAR‐T therapeutic applications, co‐expressi on of chemokine receptors to enhance tumor infiltration, and utilization ofGPCR signaling pathways to improve CAR‐T cell persistence and cytotoxic efficacy. Potential future research directions include application of AI(Artificial Intelligence) to expedite the development of GPCR antibodies, creation of precision therapies targeting GPCR complexes, modulation of GPCR dimerization networks to maintain homeostasis of membrane antigen expression, employment of nanobody platform to enhance targeting specificity, and design of GPCR allosteric modulators as molecular switches for CAR‐T cells. Additionally, this review also examines the application of specific antibodies and other immunotherapeutic approaches of GPCRs in oncology. Overall, this review aims to provide novel scientific and therapeutic perspectives for CAR‐T cell therapy in treating mutiple types of human cancers.

## Graphical abstract

Chimeric antigen receptor T cell therapy faces dual challenges of target scarcity and an immunosuppressive microenvironment in solid tumors. This review highlights how G protein‐coupled receptors can serve as both novel targets to expand the therapeutic scope and functional modules to enhance CAR‐T cell efficacy. This review also identifies key challenges and outlines future research directions in the field.

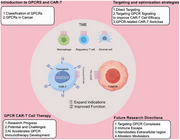

## 1 Introduction

In recent years, advances in cancer immunotherapy have garnered significant attention, highlighted particularly by the successful clinical implementation of CAR‐T therapy, which has ushered in a new therapeutic era. CAR‐T therapy is a pioneering form of immunotherapy that engineers a patient's own T cells to recognize and eliminate specific cancer cells. CAR‐T therapy has exhibited remarkable efficacy in the treatment of various hematological malignancies, though the issue of drug resistance due to single‐target limitations persists.^[^ ^1^ ^]^ The application of CAR‐T cells in solid tumors is hindered by issues such as tumor antigen heterogeneity and the immunosuppressive conditions present within the tumor microenvironment (TME).^[^ ^2^, ^3^ ^]^ GPCRs, the largest and most diverse family of transmembrane receptors, play integral roles in regulating numerous physiological processes, including cell proliferation, migration, and survival. Some of them are overexpressed in various cancers, facilitating tumor growth and metastasis.^[^ ^4^ ^]^ Furthermore, GPCRs modulate the recruitment and function of immune cells within the TME, thereby influencing the efficacy of tumor immunotherapy.^[^ ^5^, ^6^ ^]^ Therefore, GPCRs show great potential in the field of CAR‐T therapy for cancer and several other diseases. An extensive GPCR antigen library expands the scope of clinical application for CAR‐T cell therapy. Taking advantage of the functional characteristics of GPCRs in the TME and T cells can further enhance the persistence of infiltration ability, memory phenotype maintenance, and cytotoxic efficacy of CAR‐T cells, thereby comprehensively improving the efficacy of CAR‐T for the cancer therapy. Although prominent progress has been made in this field, current studies remain fragmented and lacks a systematic research framework specifically designed for cell drugstherapies. To systematically assess the landscape of this emerging field, this study comprehensively collected preclinical and clinical studies relevant to GPCR‐targeted CAR‐T cancer therapy. This review encompassed PubMed and Web of Science databases from 2000 to 2025, along with concurrent screening of the ClinicalTrials.gov database. The search strategy included three core concepts: “G protein‐coupled receptors (GPCR)”, “CAR‐T cell therapy”, and “cancer”. Retrieved records were filtered based on predefined criteria, ultimately including studies specifically focused on GPCR‐targeted CAR‐T tumor therapy while excluding those with irrelevant targets or insufficient original data. This review integrated biological characteristics of GPCR for technical requirements of CAR‐T and systematically examined the research progress, core challenges, and strategic solutions while outlining future optimization directions, aiming to establish a theoretical foundation and provide innovative perspectives for developing next‐generation GPCR‐targeted CAR‐T therapies.

## 2 GPCRs Represent an Emerging Antigen Repository for CAR‐T Therapy

**Figure 1:**
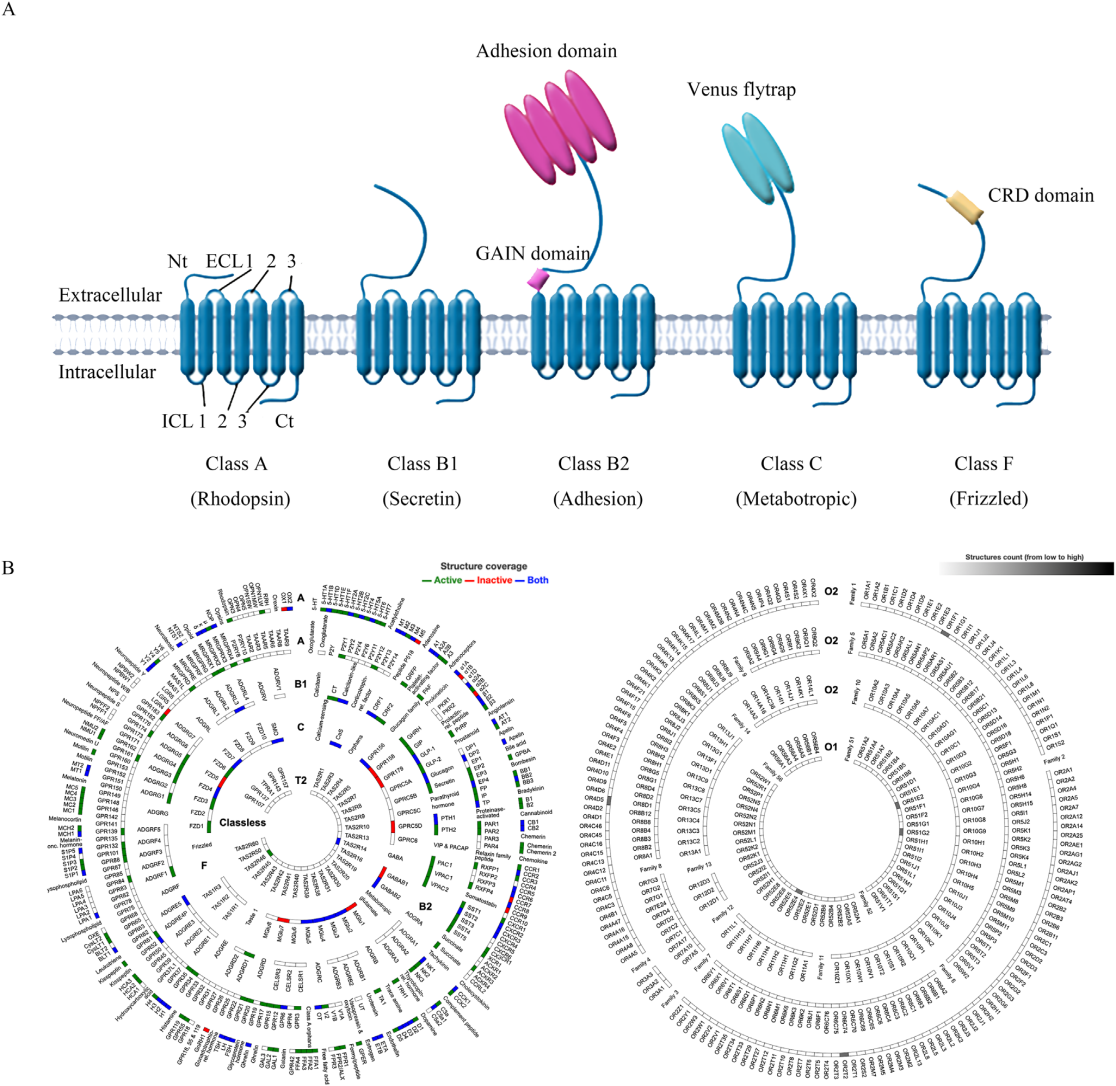
Structural features and subtype distribution of GPCR families. A) Representative structures of major GPCR families (classes A, B1, B2, C, and F). B) Proportional distribution of GPCR subtypes. Abbreviations: Nt, N‐terminus; Ct, C‐terminus; ECL, extracellular loop; ICL, intracellular loop; CRD, cysteine‐rich domain; GAIN, GPCR‐autoproteolysis inducing domain.

G protein‐coupled receptors (GPCRs), encompassing over 800 members in human genome, constitute the largest family of membrane receptors in the human genome and represent the most important class of drug targets. It is generally accepted that approximately 350 non‐olfactory receptor GPCR  are defined druggable and can serve as a substantial antigen repository for CAR‐T cell therapy. GPCRs are categorized into five main categories: A, B, C, F, and T, based on their structural and functional characteristics (**Figure** **1**A). Class A GPCRs represent the largest subclass and are responsible for sensing sensory stimuli, such as light, smell, and taste, and regulating various physiological functions, including neurotransmission, endocrine, and immune responses. Class B GPCRs are mainly divided into two subfamilies: secretins (B1) and adhesins (B2), which are involved in hormonal regulation and intracellular signaling. Class C GPCRs are essential for the proper functioning of the nervous system, especially in regulating the release of neurotransmitters.^[^ ^7^ ^]^ Class F GPCRs play a crucial role in the regulation of Wnt and Hedgehog signaling cascades. These receptors have been linked to various pathological conditions, including oncogenesis, fibrotic disorders, and processes related to embryonic development.^[^ ^8^ ^]^ Class T GPCRs (e.g., taste 2 receptors, TAS2Rs), although structurally similar to Class A, are classified as a separate category due to low sequence homology (<20%).^[^ ^9^ ^]^

Notably, GPCR database (GPCRdb) has classified odorant receptors (ORs) separately in 2025 (Figure 1B), these receptors belong to Class A GPCRs, with approximately 400 ORs in humans, accounting for about 50% of all GPCRs. Upon binding with odorant molecules, ORs initiate downstream signaling pathways, causing depolarization of olfactory neurons. Due to the characteristics of odorant molecules, which are typically low in molecular weight, highly hydrophobic, and volatile, they often require high concentrations to effectively activate ORs, making these receptors generally unsuitable as ideal drug targets for pharmaceutical development. Drugs targeting ORs, either as agonists or antagonists, need to exhibit good bioavailability throughout the body and exhibit higher affinity for their receptors than most odorants. However, recent studies have found that peptide molecules can bind to ORs with high affinity, providing a new direction for the development of drugs targeting these receptors, including CAR‐T cell therapies. Furthermore, as emerging therapeutic targets, ORs are under investigation as potential biomarkers for some malignant tumors.^[^ ^10^ ^]^

**Figure 2:**
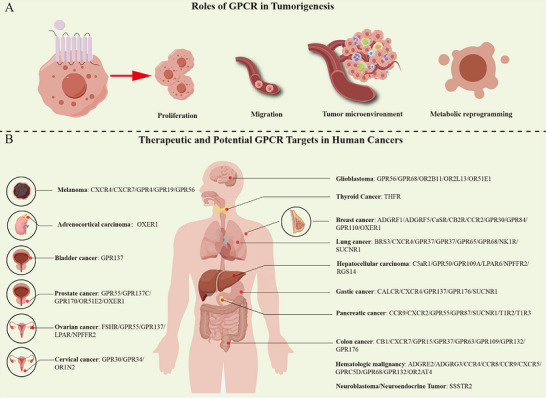
Multifunctional roles of GPCRs in tumor biology and their potentials as novel targets for CAR‐T cell therapy. A) Dysregulated expression of GPCRs in tumors influences multiple oncogenic processes, including proliferation, metastasis, tumor microenvironment remodeling, and metabolic reprogramming. B) A Systematic Summary of Potential GPCR Targets in the Majority of Human Cancers.

GPCRs are frequently dysregulated in various cancers, a process governed by a multi‐layered regulatory network encompassing genetic mutations, epigenetic alterations, and transcriptional/post‐translational modifications occurred in cancer cells.^[^ ^11^ ^]^ This aberrant expression drives multiple hallmarks of cancer, including tumor proliferation and metastasis, angiogenesis, TME remodeling, and metabolic reprogramming (**Figure** **2**A).^[^ ^12^ ^]^ Critically, the specific GPCRs overexpressed in different tumor types can activate distinct downstream signaling pathways, mediating highly context‐dependent biological effects (**Table** **1**). Consequently, A systematic overview of therapeutically promising GPCR targets across cancer types, serving as a valuable resource for developing GPCR‐directed immunotherapies is shown in Figure 2B.

**Table 1:** The role of GPCRs in cancer: Expression patterns and signaling pathways.

Cancer	GPCR	Expression	Effect	Pathways	Refs.
GBM	GPR56	↑	Tumor cell proliferation and metastasis	Wnt/β‐catenin and NF‐κB signaling pathways	[82]
	GPR68	↑	Maintenance of tumor cell survival	Inhibition of the ATF4 transcription factor	[83]
	OR2B11	↑	Strong association with immunosuppressive TAM phenotypes and mesenchymal GBM	NF‐κB/TGF‐β signaling pathways	[84]
	OR2L13	↑	Involved in neural adaptation, potentially promoting recurrence and drug resistance	–	[84]
	OR51E1	↑	Vascular remodeling and angiogenesis	–	[84]
BC	ADGRF1	HER2‐enriched BC ↑	Resistance to docetaxel	–	[85]
	ADGRF5		Regulate TME	RhoA‐ERK1/2‐C/EBPβ‐MMP8 axis	[86]
	CaSR	–	Cell migration	Gβγ‐PI3K‐mTORC2	[87]
	CB2R	↑	–	–	[88]
	CCR2		Cell proliferation, migration, and metabolism	Interacts with MET receptor tyrosine kinases	[89]
	GPR30 (tamoxifen resistance)	↑	Activate estrogen receptor signaling and promote cancer cell survival	PI3K/AKT and MEK/ERK signal pathway	[90]
	GPR84	–	Orchestrating anti‐tumorigenic macrophage polarization	Endoplasmic reticulum stress response and autophagy	[91]
	GPR110 (HER2+)	↑	Promote tumor formation, cell cycle arrest, and chemotherapy resistance	cAMP and IP1 signal pathway	[85]
	OXER1	–	Localization of macrophages and cancer cell migration	–	[92]
LC	BRS3	↑	Promoting cancer cell migration	Hippo signaling pathway	[93]
	CXCR4	↑	Cisplatin resistance	CYP1B1 upregulation	[94]
	GPR37	↑	Promoting malignant progression	TGF‐β/Smad pathway	[95]
	GPR37	↑	Promoting cell invasion, migration, and proliferation	PI3K/Akt/mTOR signaling	[96]
	GPR65 (isplatin resistance)	↑	Increased tumor growth	PKA and ERK signaling pathway	[97]
	GPR68	↑	Promotes tumor cell survival and radioresistance	Ferroptosis	[98]
	NK1R	↑	Cell proliferation and migration	ERK1/2 and Akt signaling pathway	[99]
	SUCNR1	↑	Limiting TCA cycle throughput, mitochondrial respiration, and the production of reactive oxygen species	Mediated Gαi, Akt, and ERK signaling	[100]
HCC	C5aR1	–	Inhibited HCC cell proliferation	HDAC7‐Wnt/β‐catenin axis	[101]
	GPR50	↑	Promote HCC progression	CCT6A‐induced proliferation and PGK1‐induced migration and autophagy	[102]
	GPR109A	↑	Induce M2c polarization	PKA/PPARγ/MerTK/IL‐10/TGFβ signaling pathways	[103]
	LPAR6	↑	Suppresses HCC proliferation, migration, and EMT	YAP/TAZ nuclear translocation	[104]
	NPFFR2	–	Cell proliferation and migration	RhoA/YAP axis	[105]
	RGS14	↑	Increased the proliferation, colony formation, migration, and invasion, and promoted EMT	PKA/CREB axis	[106]
ACC	OXER1	↑	Drive the formation of TNT‐like structures	Gβγ/PKCα/FARP1/Cdc42 signaling axis	[107]
GC	CALCR	↑	Promote cell proliferation, apoptosis, migration, and invasion	CALCR‐ANTXR1 axis and AKT pathway	[108]
	CXCR4	↑	Regulate TME dynamics and immune modulation	Oxidative stress and activation of the NF‐κB pathway	[109]
	GPR137	↑	Promoting cell invasion, migration, and proliferation	Hippo signaling pathways	[110]
	GPR176	–	Poor patient prognosis	Suppressing the proliferation of CD8+ T cells and facilitating immune evasion	[111]
	SUCNR1	↑	Limiting TCA cycle throughput, mitochondrial respiration, and the production of reactive oxygen species	Mediated Gαi, Akt, and ERK signaling	[100]
PC	CCR9	↑	Promotes immune evasion	–	[112]
	CXCR2	↑	Involved in cell proliferation and migration, and induced apoptosis	Chemokine signaling pathway	[113]
	GPR55	↑	Lower MEK/ERK, PI3K/AKT and YAP/TAZ signaling	mTOR signaling pathway	[114]
	GPR87	↑	Promoting the proliferation, angiogenesis, and increased resistance to gemcitabine‐induced apoptosis	NF‐κB signaling pathway	[115]
	SUCNR1	↑	Limiting TCA cycle throughput, mitochondrial respiration, and the production of reactive oxygen species	Mediated Gαi, Akt, and ERK signaling	[100]
	T1R2/T1R3	↑	Induced epigenetic reprogramming modeling of tumorigenesis and metastasis	MMP‐9 and Neu‐1	[116]
CRC	CB1	–	Orchestrating intercellular conduits to enhance metabolic drivers to stage migratory intercellular communication	TNT communications	[117]
	CXCR7	↑	Promoting EMT and invasion	YAP1 nuclear translocation	[118]
	GPR15	↑	Migration and invasion	–	[119]
	GPR37	↑	Modulates lipid metabolism	mMAPK‐p38‐SCD1	[120]
	GPR63	↑	Promoting CRC cell migration	JAK2/STAT3 pathway	[121]
	GPR109	–	Inhibited glucose transport and glycolysis	GPR109a‐AKT signaling pathway	[122]
	GPR132	–	Inhibits the maturation and cytotoxic function of NK cells	CSK/ZAP70/NF‐κB signaling axis	[123]
	GPR176	↑	Promoting the tumorigenesis and progression	GPR176/GNAS complex inhibits mitophagy via the cAMP/PKA/BNIP3L axis	[124]
PCa	GPR55	–	Resist proliferation and promote apoptosis	–	[125]
	GPR137C	↑	Promotes tumor microenvironment remodeling by enhancing immune cell infiltration	–	[126]
	GPR170	–	Regulate cell growth, differentiation, and motility	Testosterone‐induced calcium response with the non‐AR pathway	[127]
	OR51E2	↑	Inhibit aggressive cancer phenotype	STAT3 signaling pathway	[128]
	OXER1	–	Localization of macrophages and cancer cell migration	–	[92]
OC	GPR55	↑	Inhibiting the pro‐angiogenic LPI/GPR55 pathway	LPI/GPR55 pathway	[129]
	GPR137	↑	Stimulates cell migration abilities	PI3K/AKT pathway	[130]
	GPR137	↑	Stimulates cell proliferation, migration, invasion, and colony formation	GPR137‐RAB8A‐HH cascade	[131]
	LPAR	↑	Stimulates cell proliferation and migration	UCA1‐miR‐let7a axis	[132]
	NPFFR2	↑	Stimulates EOC cell invasion	ERK1/2‐MMP‐9 axis	[133]
BC	GPR137	↑	Promotes growth and proliferation	Blocks the G0/G1 and S phases of the cell cycle	[134]
CC	GPR30	↑	Induce apoptosis, inhibit migration, and cause G1 phase arrest	–	[135]
	GPR34	↑	Cell proliferation	–	[136]
	OR1N2	–	Activation induces cell fusion of cervical cancer cells	Non‐canonical olfactory pathway	[137]
NB	SSTR2	↑	–	–	[138]
NET	SSTR2	↑	–	–	[139]
Melanoma	CXCR4/CXCR7	↑	Promote the chemotaxis and chemokines of melanoma cells	SDF1/CXCR4/CXCR7 interaction	[140]
	GPR4	↑	Promote the migration of melanoma cells in the tumor periphery	The pH‐dependent migration	[141]
	GPR19	↑	Increase the proliferation, migration, and invasive capabilities	–	[142]
	GPR56	↑	Promote amoeboid‐like morphological change and increased IL‐6 production	Rho‐ROCK‐MLC and JAK‐STAT3 pathways	[143]
HM	ADGRE2	↑	Supports leukemia stem cell self‐renewal and leukemogenesis	MEK/AP1/DUSP1 axis	[144]
	ADGRG3	↑	Neutrophil degranulation and GPCR signaling	–	[145]
	GPR68	↑	Promoting cell survival, chemoresistance	Ca2+/Calcineurin pro‐survival pathway, mediated by glucose metabolic symbiosis	[146]
	GPR132	–	Cell differentiation	Activation of GPR132	[147]
	GPRC5D	↑	–	–	[15]
	OR2AT4	↑	Induce apoptosis differentiation and reduction of proliferation	p38‐MAPK and p44/42‐MAPK phosphorylation	[148]

## 3 The Current Landscape of GPCR CAR‐T Cell Therapy

CAR‐T cells are engineered by incorporating the genetic cassette that encodes the CAR into T lymphocytes extracted from the peripheral blood of patients. This modification is achieved by either viral vectors, such as lentiviruses or retroviruses, or non‐viral approaches, including electroporation, transposonor CRISPR systems. The transmembrane CAR facilitates the ability of T cells to distinctly identify and eradicate cancerous cells in vivo. CAR typicallyincludes three functional domains. The extracellular domain typically comprises a specific type of antibody that plays a crucial role for the specific recognition of antigens located on the surfaces of tumor cells. The transmembrane segment plays a vital role in anchoring the CAR to the membrane of T‐cells. Typically, the costimulatory domain encompasses the CD3ζ chain alongside costimulatory molecules such as CD28 or 4‐1BB, which collectively facilitate the activation of T‐cell proliferation, enhance cytotoxicity, and stimulate the secretion of essential cytokines.^[^ ^13^ ^]^ Kymriah, a CAR‐T drug that targets CD19, has showcased remarkable effectiveness to  treate B‐cell acute lymphoblastic leukemia (B‐ALL), thus achieving recognition as the inaugural CAR‐T therapy with FDA approval in 2017.^[^ ^14^ ^]^ Subsequently, CAR‐T therapy has also shown encouraging clinical evidence in other hematological malignancies, including multiple myeloma (MM). As of 2025, the FDA has approved seven CAR‐T cell products, all of which target single‐pass transmembrane proteins. However, despite the widespread evidence of abnormal GPCR expression in tumors and their critical role in regulating the immune microenvironment, no CAR‐T therapy targeting seven‐transmembrane GPCRs has been approved. GPCR‐targeted CAR‐T own unique advantages. First, the vast diversity of GPCRs provides a rich antigen library for CAR‐T, enabling coverage of a broad range of indications for both hematologic and solid tumors. For patients resistant to existing targets, GPCR‐targeted strategies can prevent treatment failure due to single‐target resistance or provide alternative treatment options.^[^ ^15^ ^]^ Second, the topological features of GPCRs (e.g., N‐terminal glycosylation sites, extracellular loop variability) enable the design of multivalent antigen recognition domains, significantly enhancing the antigen recognition specificity of CAR‐T through spatial complementarity. Finally, GPCR‐mediated cancer‐related pathways can be engineered into CAR‐T designs, simultaneous recognition and blockage of GPCR target could enhance anti‐tumor efficacy by improving CAR‐T cell function or modifying the TME.

Preclinical studies of GPCR‐CAR‐T (**Table** **2**) have covered multiple targets and diseases, demonstrating dual potential in treating both hematologic and solid tumors. Among the array of clinical trials focusing on GPCR‐targeted CAR‐T therapies, the advancement of GPRC5D‐targeted CAR‐T cells for MM has been particularly notable. While B‐cell maturation antigen (BCMA)‐targeted CAR‐T therapy has significantly enhanced treatment outcomes for MM, a considerable number of patients face relapse attributed to antigen escape mechanisms. GPRC5D, an innovative target independent of BCMA expression, is highly expressed in MM cells, providing a critical breakthrough for overcoming drug resistance. In 2022, the New England Journal of Medicine published the initial clinical results of the GPRC5D‐targeted CAR‐T therapy MCARH109.^[^ ^15^ ^]^ Among patients who had previously received BCMA CAR‐T therapy, this treatment demonstrated a 71% objective response rate (ORR) and a 35% complete response rate (CR), with efficacy independent of baseline BCMA expression levels. Updated data after a median follow‐up of 37 months further confirmed its long‐term safety profile, with no new serious adverse events reported during this period. Notably, among 10 patients resistant to BCMA‐targeted therapy, 70% still achieved complete response, with a median duration of response (mDOR) of 8.6 months, indicating sustained efficacy in this refractory population.^[^ ^16^ ^]^ Subsequent clinical studies have consistently demonstrated the broad efficacy of GPRC5D CAR‐T: interim analysis of CC‐95266 showed an ORR of 86%,^[^ ^17^ ^]^ a Chinese clinical trial reported an ORR of 91%,^[^ ^18^ ^]^ while preliminary data from both OriCAR‐017 and CT071 indicated ORRs of 100%.^[^ ^19^ ^]^ None of these efficacy outcomes showed significant correlation with patients' prior exposure to BCMA‐targeted therapies. Regarding safety, the main adverse events associated with GPRC5D CAR‐T were Grade 1–2 cytokine release syndrome and reversible neurotoxicity, with some patients experiencing skin/nail‐related toxicities or transient olfactory disturbances. Only a small proportion of patients developed ≥ Grade 3 adverse events. Building on the significant efficacy and manageable safety profile of this monotherapy, researchers are actively advancing the development of dual‐targeting CAR‐T therapies. Two GPRC5D/BCMA bispecific CAR‐T products have now entered Phase I clinical trials. Among these, YK‐CAR‐069 demonstrated an 86% ORR in 21 patients with relapsed/refractory multiple myeloma (R/R MM), with the optimal dose cohort achieving 92% ORR. Another trial reported 100% ORR among 9 R/R MM patients with extramedullary infection, with all cases of cytokine release syndrome being Grade 1–2 and no severe neurotoxicity observed. Detailed efficacy and safety profiles for all therapies are summarized in Table 3. GPRC5D CAR‐T is poised to overcome current therapeutic limitations and emerges as a cornerstone therapeutic approach for multiple myeloma. Further exploration of its potential in solid tumors will subsequently promote broader application of GPCR‐targeted CAR‐T therapies.

**Table 2:** Summary of Preclinical Studies on GPCR‐Targeted CAR‐T Cells and GPCR Modules for CAR‐T Enhancement.

Target	Indications	Signaling Domain	Additional Engineering	Refs.
ADGRE2	AML	4‐1BB–CD3ζ	Co‐expression of CLEC12A	[149]
B7‐H3	NSCLC	CD28‐CD3ζ/4‐1BB‐CD3ζ	Co‐expression of the CCL2 receptor CCR2b	[51]
CD19/BCMA	Leukemia	4‐1BB‐CD3ζ	Add a C3aR co‐stimulatory domain	[58]
CCR4	T cell malignancies	4‐1BB‐CD3ζ	CCR4‐depleted in CAR‐T cells	[33]
CCR8	T‐ALL	CD28‐CD3ζ	–	[34]
CCR9	T‐ALL	4‐1BB–CD3ζ	Co‐expression of CD1a	[150]
CCR9	T‐ALL	4‐1BB‐CD3ζ	Incorporate a suicide gene into CAR‐T cells	[35]
CCR10	Myeloma	4‐1BB‐CD3ζ	CCR10‐depleted in CAR‐T cells	[151]
CXCR5	B‐NHL	CD28‐CD3ζ	–	[36]
EGFR	NSCLC	4‐1BB‐CD3ζ	Co‐expression of CXCR5	[52]
EGFRvIII	BC	CD28‐CD3ζ	Co‐expression of IL‐15/IL‐18 and CXCR2	[152]
EpCAM	PC	CD28‐CD3ζ	Co‐expression of CCR8 and DNR	[153]
FSHR	OC	4‐1BB‐CD3ζ	–	[154]
GD2	Melanoma; NB	4‐1BB‐CD3ζ	Co‐expression of the CCR2b and IL‐7	[155]
GPC3	HCC	4‐1BB‐CD3ζ	Co‐expression of CXCR2	[156]
GPRC5D	MM	4‐1BB‐CD3ζ	–	[17]
MSLN	PC	CD28‐CD3ζ	Co‐expression of CXCR6	[157]
OR2H1	NSCLC; OC	4‐1BB‐CD3ζ	–	[37]
SSTR	NET	CD28‐CD3ζ	–	[41]
TSHR	TC	CD28‐4‐1BB‐CD3ζ	–	[38]

**Table 3:** Outcomes of GPRC5D CAR‐T clinical trials in MM.

Agent	Patients number	ECD	ORR (Prior BCMA therapy)	Efficacy (All)	Adverse event (Grade ≥ 3)	CTID	Phase	Refs.
MCARH109	17	scFv	70%	ORR (71%), CR (35%)	CRS (6%), ICANS (6%)	NCT04555551	I	[15]
OriCAR‐017	10	VHH‐VHH	100%	ORR (100%), CR (60%)	CRS (0%), ICANS (0%)	NCT05016778	I	[19]
CC‐95266	17	scFv	67%	ORR (86%)	CRS (0%), ICANS (0%)	NCT04674813	I	[158]
GPRC5D CAR T	33	scFv	100%	ORR (91%), CR (64%)	CRS (0%), ICANS (3%)	ChiCTR2100048888	II	[159]
CT071	20	–	100%	ORR (100%), CR (50%)	CRS (0%), ICANS (5%)	NCT05838131	I	[160]
‌YK‐CAR‐069	21	scFv	–	ORR (86%), CR/sCR (62‐75%)	CRS (71%), ICANS (5%)	NCT05509530	I	[161]
BCMA/GPRC5D CAR T	9	scFv	–	ORR (100%), CR/sCR (44.4%)	CRS (66.7%)	2024EC1‐ky033	I	[162]

## 4 Evaluation, Challenges and Optimization Strategies for GPCRs as CAR‐T Targets

The development of CAR‐T cell therapies targeting GPCRs faces unique challenges distinct from those of small‐molecule GPCR modulators, necessitating a tailored evaluation framework that integrates conventional CAR‐T experience with GPCR‐specific biological profile. While promising preclinical study have been carried out for several GPCR targets, the translational potential for clinical intervention still requires rigorous examination. The clinical development of GPRC5D‐targeted CAR‐T therapies for treating multiple myeloma has provided a classic benchmark for this emerging class of novel targets. Generally, an ideal GPCR target for CAR‐T should exhibit high and specific expression on tumor cell surface, strict absence in vital organs, and restricted low expression in dispensable tissues. To mitigate the on‐target, off‐tumor (OTOT) toxicity, GPCR CAR‐T therapies also should primarily target selective GPCR with high tumor specificity, strict absence in vital organs, and or restricted low expression in non‐essential tissues, ensuring any potential toxicities manifest as controllable, reversible clinical presentations without critical organ dysfunction. Therefore, GPCR target validation should commence with comprehensive cross‐tissue expression profiling using RNA‐seq, immunohistochemistry, in situ hybridization, and flow cytometry approaches. Furthermore, the expression gradient between tumor and normal tissues serves as a critical parameter for evaluating such toxicity risks. GPRC5D exemplifies these ideal characteristics, with off‐target toxicity primarily confined to self‐limiting mild reactions in skin, nails, or oral mucosa that are readily manageable and clinically acceptable. Data show GPRC5D mRNA levels in multiple myeloma cells are 1000‐fold and 500‐fold higher, respectively, than normal plasma cells and peripheral blood B cells. This marked expression gradient establishes a benchmark for screening other GPCR targets, underscoring the importance of expression differentials for target selection.^[^ ^20^ ^]^ Additionally, as seven‐transmembrane proteins, GPCRs have limited extracellular domains comprising three loops and an N‐terminus, potentially reducing immune synapse efficiency. Thus, the antigen density required for effective GPCR CAR‐T activation may exceed that of conventional targets. To address this, high‐affinity CARs could enhance the recognition of cells with low antigen density, but this may narrow the therapeutic window by increasing cross‐reactivity risks with normal tissues or homologous GPCRs.^[^ ^21^ ^]^ A recent Nature study described a redirected proximity labeling strategy that establishes a new paradigm for locally remodeling immune targets. This technique non‐invasively generates high‐density synthetic antigens (fluorescent clusters) in vivo by replacing poorly recognizable native targets (e.g., low‐density GPCRs) with defined, stable neoantigens amenable to efficient targeting. This enables subsequent T‐cell engagers or CAR‐T therapies targeting these neoantigens to achieve maximal eliminating efficacy, providing a versatile platform against heterogeneity and low density issues of antigens.^[^ ^22^ ^]^ Given the high homology among GPCR family members, ensuring targeting specificity for CAR‐T cell therapies is crucial. Subsequent sections will describe the utilization of high‐specificity nanobody to overcome cross‐reactive issues.^[^ ^23^ ^]^ Clinical observations have documented disease relapse due to antigen loss following GPRC5D CAR‐T therapy, indicating tumor antigen heterogeneity as a key resistance mechanism. Consequently, developing dual‐targeting CAR‐T therapies against antigens such as GPRC5D and BCMA has emerged as an alternative strategy to address this challenge. Furthermore, given the prevalence of splice variants of some GPCRs, early CAR development requires integrated analysis of all known variants and their tumor expression profiles using bioinformatic databases and tumor genomic sequencing datasets. The ideal CAR epitope should reside within structurally conserved extracellular regions shared across all oncogenic splicing variants. Although bispecific CARs can engage multiple epitopes, their complex manufacturing and high costs constrain clinical translation, making the selection of universally conserved epitopes the primary goal. For GPCRs with endogenous ligands, epitopes outside ligand‐binding domains should be prioritized to prevent interference from ligand competition or steric hindrance. In addition, functional validation must also rigorously confirm that CAR binding does not aberrantly activate downstream signaling in tumor or T cells, which is essential for therapeutic safety.

## 5 Artificial Intelligence Accelerates Antibody Development for GPCRs

GPCRs play crucials th role in physiological processes within the human body, and disruptions in the regulation of GPCR‐related intracellular signaling pathways, or the targeting of body cells by exogenous pathogenic factors through GPCRs, can result in the development of multiple diseases. As a result, GPCRs are acknowledged as one of the most frequently utilized therapeutic targets. In 2018, annual sales of GPCR‐targeted medications surpassed $114 billion. In 2019, five out of the 20 approved innovative drugs targeted GPCRs.^[^ ^24^ ^]^ Presently, GPCR medications approved by the FDA make up almost 40% of all approved drugs,^[^ ^25^ ^]^ of which small molecule drugs account for 92%, peptide drugs 5%, protein drugs 2%, and only two are antibodies. Specifically, they are Mogamulizumab (targeting CCR4) for T‐cell lymphoma treatment, and Erenumab (targeting CGRPR) for migraine. Furthermore, the monoclonal antibodies fremanezumab and galcanezumab act by binding to and neutralizing the ligand of CGRP, thereby blocking its receptor interaction, rather than by directly targeting the GPCR itself. By April 2025, >170 GPCR antibody pipelines globally are in preclinical to Phase III stages, covering 76 targets, mainly concentrating on cancer, metabolic disorders, and immune inflammation.^[^ ^26^, ^27^ ^]^ Aside from the marketed antibody drugs that target GPCRs, there are two drugs that have entered Phase III clinical trials or are nearing market release: Leronlimab that targets CCR5 and is indicated for HIV infection, TNBC, and NASH; and GMA102 (Glutazumab) that targets GLP‐1R and is indicated for type 2 diabetes and obesity. The antibody targets with the highest number of ongoing pipelines include CCR8, CXCR4, C5aR1, GCGR, CXCR5, CCR5, GLP‐1R, CB1, and CCR7.

However, the development of GPCR‐targeted CAR‐T faces core challenges in antibody development. First, the difficulty of reconstruct native conformations of GPCRs in vitro, as their transmembrane nature and structural instability result in low efficiency in functional antigen screening. Secondly, the structural diversity of the GPCR family (e.g., differences in transmembrane region conservation, extracellular loop variability) necessitates subtype‐specific optimization in antibody design, particularly in terms of steric hindrance and epitope accessibility in the ligand‐binding domain. Additionally, the complex ligand‐specific recognition mechanisms of GPCR subtypes (e.g., biased signaling) demand antibodies with both high affinity and precise signaling pathway regulation capabilities. More critically, as central hubs in cellular signaling networks, GPCR dysfunction can lead to systemic side effects, further requiring antibodies to achieve a triple balance of target selectivity, functional controllability, and biosafety, thereby increasing the difficulty of developing highly efficient and specific antibody molecules.^[^ ^28^, ^29^, ^30^ ^]^

Traditional antibody development technologies, such as animal immunization and in vitro phage display, have long been hampered by limitations including low efficiency, poor stability, and challenges in clinical translation. Artificial intelligence(AI) offers innovative solutions to overcome these constraints. In antibody design platform, deep learning could integrate multidimensional biological data to accurately identify key design parameters, enabling not only the prediction of how mutations affect antibody affinity and specificity but also the direct determination of three‐dimensional protein structures from the primary amino acid sequences. In the field antibody development of GPCRs, multiple AI models have demonstrated substantial potentials. AlphaFold‐Multimer has successfully predicted nanobodies targeting MRGPRX2 through virtual screening, with experimental confirmation of high affinity and optimal functional activity.^[^ ^31^ ^]^ Concurrently, the JAM framework reported in Science has improved the success rate of antibody design for CXCR7 and CXCR4 by more than 60‐fold.^[^ ^32^ ^]^ Particularly noteworthy is the breakthrough in agonist antibody development achieved by this approach, which successfully generated the first CXCR7 agonistic antibody demonstrating efficacy comparable to its natural ligand. These advancements underlined the transformative capability of AI technologies in antibody development, especially for multiple transmembrane receptors.

## 6 GPCRs‐Targeting CAR‐T Therapies

**Figure 3:**
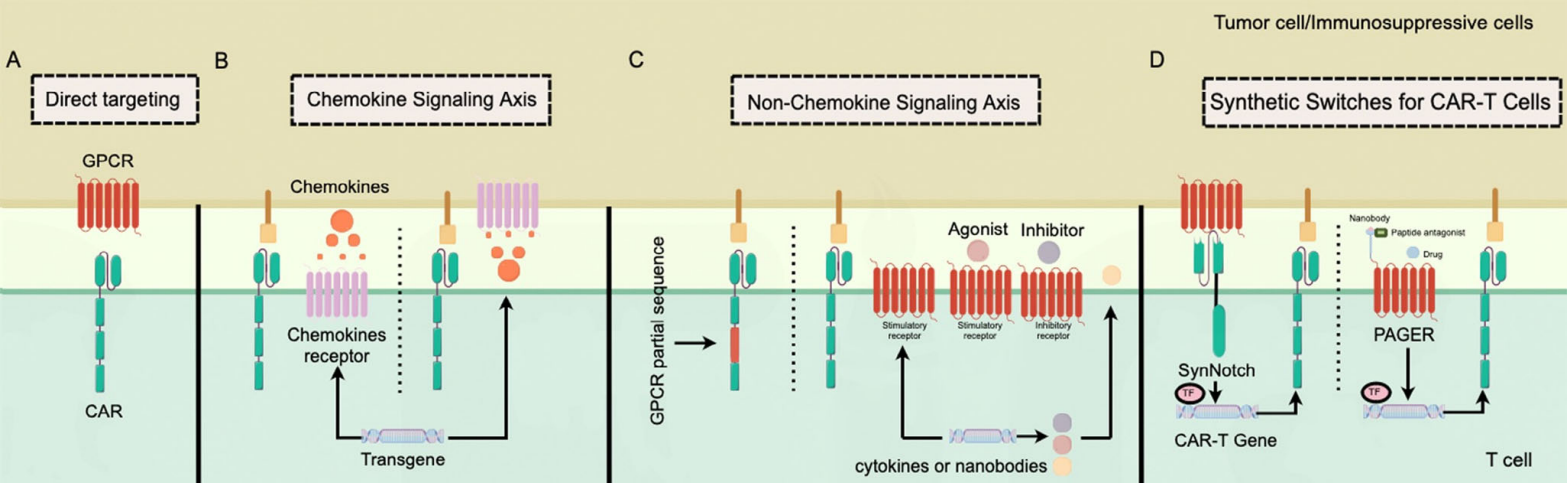
From Direct Targeting to Functional Enhancement: The Dual Roles of GPCRs in CAR‐T Cell Therapy. A) Direct targeting: CAR‐T cells are engineered to directly target GPCRs on the surface of tumor cells. B) Targeting the Chemokine Signaling Axis: CAR‐T cells co‐express chemokine receptors or chemokines that guide them to the tumor site. C) Targeting the Non‐Chemokine Signaling Axis: Incorporating GPCR‐derived domains as co‐stimulatory elements can enhance specific functional properties of CAR‐T cells (Left); Overexpression of T cell‐potentiating GPCRs in CAR‐T cells modulates their proliferation and differentiation through activation of downstream signaling pathways; Engineering CAR‐T cells to secrete modulators such as antagonists, agonists, cytokines, or nanobodies enables remodeling of the tumor microenvironment and enhances therapeutic efficacy (Right). D) GPCR‐Related Synthetic Switches for CAR‐T Cells: When a cell surface antigen (such as a GPCR) binds to the scFv/nanobody domain of a SynNotch receptor, it induces the release of the intracellular transcription factor domain into the nucleus. This engineered transcription factor then binds to a specific, user‐defined promoter (e.g., Gal4 UAS), which controls the CAR transgene, thereby driving its high‐level and specific expression (Left). The binding of a soluble target antigen to the nanobody‐based autoinhibition module relieves the steric hindrance, activating the native GPCR.The activated GPCR triggers its intrinsic downstream signaling pathway, leading to the production of second messengers (e.g., cAMP). This leads to activation and expression of the CAR gene under the control of cAMP response elements (Right). By Figdraw: APSII09009.

The most common targeting approach involves designing the antigen recognition domain of CAR to directly target GPCR‐expressing cells using antibodies/ligands (**Figure** **3**A). Despite the encouraging efficiency exhibited by CAR‐T cells in the clinical intervention of B‐cell leukemia, the utilization of CAR‐T therapy for T‐cell lymphoma encounters obstacles primarily attributed to the limited availability of suitable targets. Research has indicated that CAR‐T cells designed to target CCR4 and CCR8 are capable of efficiently eradicating cells associated with adult T‐cell leukemia/lymphoma.^[^ ^33^, ^34^ ^]^ CCR9 is highly expressed in 70% of T‐cell acute lymphoblastic leukemia (T‐ALL) patients, and research has shown that CCR9 CAR‐T cells demonstrated strong killing efficacy even at low antigen density.^[^ ^35^ ^]^ Since CAR‐T cells also express corresponding targets, the deletion of respective receptors before CAR‐T cell preparation can mitigate self‐targeting‐induced apoptosis. This strategy increases the proportion of Th1 and CD8+ T cells, effectively targeting and killing CCR4‐expressing cells. CAR‐T cell therapy for B‐cell malignancies and MM is challenged by significant target homogeneity, which leads to issues such as antigen escape and drug resistance. This highlights the critical need for identifying novel targets and developing multi‐target strategies. CXCR5 is physiologically expressed in mature B cells and T follicular helper (Tfh) cells and is highly expressed in lymph node B‐cell non‐Hodgkin lymphoma (B‐NHL). Compared to CD19 CAR‐T cells, CXCR5 CAR‐T cells are more effective in eliminating B‐NHL cells and supportive Tfh cells in vitro. In xenograft mouse models, CXCR5 CAR‐T cells effectively inhibit lymphoma growth, and in allogeneic mouse models, they selectively deplete endogenous and malignant B cells and Tfh cells without causing off‐target toxicity.^[^ ^36^ ^]^

GPCRs demonstrate specific and diverse expression patterns in solid tumors, offering new opportunities for CAR‐T therapy in this challenging area. OR2H1 is widely expressed in solid epithelial tumors and normal human testicular tissue. CAR‐T cells targeting OR2H1 can specifically recognize and eliminate OR2H1+ cancer cells, offering potential therapeutic benefits for various epithelial cancers.^[^ ^37^ ^]^ Thyroid‐stimulating hormone receptor (TSHR) is highly expressed in several thyroid cancers, including follicular thyroid cancer, cervical lymph node metastasis, and radioiodine‐resistant diseases. TSHR CAR‐T cells (K1‐70), developed by Li et al., demonstrated significant anti‐tumor effects in vitro and showed therapeutic efficacy in advanced thyroid cancer without significant toxicity.^[^ ^38^ ^]^ LGR5 (GPR49), widely distributed in human tissues such as the breast, hair follicles, eyes, reproductive organs, and gastrointestinal tract, is a potential biomarker for various cancer stem cells (CSCs). LGR5 CAR‐T cells target CSCs, inhibiting the tumor's ability to regenerate, thus continuously suppressing tumor growth and preventing recurrence, particularly in colorectal cancer patients.^[^ ^39^ ^]^ Neuroendocrine tumors often overexpress somatostatin receptors (SSTRs). Octreotide, a synthetic somatostatin analog, is utilized as the extracellular domain of CAR‐T cells to target and eliminate neuroendocrine tumor cell lines overexpressing SSTR2/SSTR5 in mice without causing obvious off‐target toxicity. Follicle‐stimulating hormone receptor (FSHR) is primarily expressed in adult ovarian granulosa cells and testicular Sertoli cells. Studies by Radu and colleagues show that FSHR is commonly expressed in tumor‐associated vasculature across various tumor types but not in the surrounding healthy vasculature. The natural ligand FSH is utilized as the extracellular domain of FSH CAR‐T cells to effectively mediate the killing of FSHR‐expressing tumor cells in both human and mouse models. Additionally, FSH‐CAR could also inhibit tumor growth in syngeneic and xenograft tumor‐bearing mouse models.^[^ ^40^, ^41^ ^]^ Elevated levels of OR7C1 are associated with unfavorable outcomes in colorectal cancer patients, and specific cytotoxic T lymphocytes (CTLs) can identify and eliminate cancer‐initiating cells (CICs) in colorectal cancer, highlighting OR7C1 as a promising GPCR target for CAR‐T cell therapy.^[^ ^42^, ^43^ ^]^ In addition to the direct eradication of tumor cells, the strategy of targeting immunosuppressive cellular populations within the TME—such as the depletion of regulatory T cells (Tregs)—can significantly augment the tumoricidal efficacy of CAR‐T cells.^[^ ^33^, ^36^ ^]^

## 7 Targeting GPCR Signaling to Improve CAR‐T Cell Efficacy

### 7.1 The GPCR‐Mediated Tumor Microenvironment

GPCRs are extensively distributed across diverse cell types within the tumor microenvironment and function as critical hubs regulating tumor growth and metastasis, profoundly influencing its immunological characteristics. Beyond directly targeting tumor cells expressing GPCRs, engineering strategies that modulate CAR‐T cell responses to GPCR signaling represent another effective approach to enhance their antitumor efficacy. The aberrant expression of GPCRs in tumor cells directly drives malignant phenotypes such as proliferation, survival, migration, and angiogenesis. Tumor cells hijacked chemokine‐mediated migratory activity by abnormally expressing chemokine receptors, thereby achieving distant metastasis. Notably, CXCL12/SDF‐1, which serves as the ligand for CXCR4, exhibits markedly elevated expression levels in prevalent metastatic sites, including the lymph nodes, lungs, bone marrow, and liver.^[^ ^44^ ^]^ In breast cancer, CXCR4 overexpression promotes metastasis by activating the P‐REX1‐Rac1 pathway, while in basal‐like breast cancer, CXCR4 coupled to G12/13 drives metastatic progression in a RhoA‐dependent manner.^[^ ^45^ ^]^ Moreover, additional chemokine receptors, including CCR7, CCR10, and GPR116, have been identified as playing a significant role in the modulation of cancer cell survival, proliferation, invasion, and migration.^[^ ^46^, ^47^ ^]^ GPCRs and their associated ligands play critical roles in the regulation of angiogenesis. This process occurs through two primary mechanisms: Initially, this process promotes the direct stimulation of endothelial cell proliferation. Additionally, it indirectly enhances the release of VEGF from multiple sources, such as stromal cells, immune cells, and neoplastic cells.

Furthermore, GPCRs profoundly shape the TME and may restrict the anti‐tumor immunity by regulating the recruitment and function of various immune cells within the TME. For instance, GPR65 is expressed in tumor‐associated macrophage, that inhibit antitumor immune activities in an acidic microenvironment. Accordingly, GPR65 small molecule inhibitor PTT‐3213 could significantly increase the number of CD8+ T cells and NK‐T cells in the TME and synergize with PD‐1 antibody in mouse tumor bearing model. CCR8 is expressed on Treg cells, and tumors recruit Tregs via the CCL1‐CCR8 axis. these cells subsequently suppress immune cell function through TGF‐β secretion, establishing this axis as a critical immunosuppressive hub. Tregs secrete TGF‐β, which suppresses immune cell activity. GPCRs are also broadly expressed in other immune cells within the TME, and their signaling pathways collectively form a complex immunoregulatory network. Finally, GPCRs are also overexpressed in stromal cells of the TME, such as fibroblasts, where they exert complex regulatory functions.For instance, the role of GPR132 can be either pro‐tumorigenic or anti‐tumorigenic depending on the cellular origin of the tumor.^[^ ^48^, ^49^ ^]^

Building upon the pivotal functions of GPCRs in the tumor microenvironment, the following section elaborates on two principal strategies. The first strategy addresses the “spatial localization” of CAR‐T cells via chemokine receptors to enhance their tumor infiltration capacity. The second strategy employs non‐chemokine GPCRs to modulate their “functional state” to overcome immunosuppression and improve persistence.

### 7.2 Enhancing CAR‐T Cell Tumor Infiltration Through THE Modulation of Chemokine Signaling Axis

A key obstacle for CAR‐T cells for immunotherapy is their limited capacity to penetrate solid tumors. The chemokine/chemokine receptor axis related to cancer cell growth and metastasis offers a prospective approach to improve migration efficiency of CAR‐T cells. However, the “mismatch” between chemokines on tumor and immune cells and their receptors often results in low infiltration efficiency and suboptimal anti‐tumor effects. Chemokine receptors are typically categorized within the GPCR family. To tackle this challenge, an effective strategy involves the co‐expression of chemokine receptors that correspond to the chemokines released by tumor cells on CAR‐T cells. This strategy aims to rectify the existing “mismatch” and improve the responsiveness of CAR‐T cells to tumor‐specific chemokines, as illustrated in Figure 3B. For instance, Hodgkin lymphoma (HL) cells produce CCL17 and CCL22. CAR‐T cells that co‐express CCR4 can migrate toward HL cells, enhancing tumor control efficacy.^[^ ^50^ ^]^ In the context of non‐small cell lung cancer (NSCLC), tumor tissues exhibit a significant upregulation of the chemokine CCL2. The concurrent expression of its receptor, CCR2, on CAR‐T cells enhances their capacity of tumor infiltration by approximately threefold. Furthermore, malignant pleural mesothelioma is characterized by elevated levels of CCL2 secretion, and CAR‐T cells that co‐express CCR2b demonstrate an impressive 12.5‐fold augmentation in their tumor infiltration potential.^[^ ^51^ ^]^ Epidermal growth factor receptor (EGFR) CAR‐T cells co‐expressing CXCR5 can promote migration toward CXCL13‐expressing NSCLC tumors and minimize potential off‐tumor, on‐target toxicity of EGFR‐CAR‐T.^[^ ^52^ ^]^ The concurrent release of IL‐7 from CAR‐T cells modified to express CXCR5 significantly boosts their therapeutic efficacy in the context of osteosarcoma treatment.^[^ ^53^ ^]^ Hepatocellular carcinoma highly expresses CXCL13, and CAR‐T cells co‐expressing its ligand CXCR5 achieve complete remission in CDX mouse models. CXCL16 is highly expressed in pancreatic cancer cells and tumor‐infiltrating immune cells. CAR‐T cells that co‐express CXCR6 exhibit improved accumulation within tumors, prolonged anti‐tumor efficacy, and increased survival rate in murine models. Besides the co‐expression of chemokine receptors, CAR‐T cells possess the capability to secrete chemokines and interact with chemokine receptors present on tumor cells. For instance, CAR‐T cells that release both IL‐7 and CCL19 have the potential to modify the TME, thereby enhancing T cell recruitment and improving survival rates.^[^ ^54^ ^]^ In conclusion, GPCRs as targets for CAR‐T cells demonstrate great immense potential. By targeting specific GPCRs, more precise CAR‐T cells can be designed to increase their homing capacity to tumors while reducing toxicity to normal tissues, potentially improving the efficacy of cancer immunotherapy.^[^ ^55^ ^]^ Further research requires i more effective or cocktail medical options for specific cancer type. Beyond CAR‐T cells, CAR‐NK cells offer greater application potential due to their innate killing ability, lower cytotoxicity, and donor matching advantages. For instance, co‐expressing CCR7 on CD19 CAR‐NK cells has been shown to improve homing to lymph node chemokines in vitro and in vivo, resulting in effective tumor control.^[^ ^56^ ^]^ Similarly, co‐expressing CXCR1 on GD2 CAR‐NK cells has demonstrated enhanced tumor infiltration and anti‐tumor responses.^[^ ^57^ ^]^

### 7.3 Enhancing CAR‐T Efficacy with Non‐Chemokine GPCR Signaling Modules

The regulatory role of GPCR signaling pathways in T cell activation and differentiation provides a rationale for their integration as functional signaling modules in CAR‐T engineering, offering new avenues to enhance antitumor efficacy. A representative approach involves functional expression of complement receptor C3aR (aa161‐340) in bispecific CAR‐T cells, which has been shown to specifically expand Th17 and central memory T cell subsets, enhancing leukemic cell clearance while maintaining T cell persistence (Figure 3C).^[^ ^58^ ^]^ Fourth‐generation CAR‐T cells, commonly termed “TRUCK” cells, can constitutively or transiently express transgenic proteins to simultaneously target multiple GPCR pathways involved in tumor progression, thereby enhancing antitumor efficacy and treatment durability (Figure 3C). The targets and associated pathways listed in Table 1 provide key references for the design and application of TRUCK CAR‐T cells. A highlighted illustration demonstrates the ability of dopamine to promote the differentiation of CD8+ T cells into tissue‐resident memory T cells via the activation of the DRD5 receptor, thereby enhancing anti‐tumor immunity and yielding improved outcomes of patients. DRD5‐co‐expressing CAR‐T cells designed based on this principle demonstrate significantly enhanced killing efficacy in colorectal cancer models.^[^ ^59^ ^]^ The A2A receptor binds to adenosine, suppresses immune cell function and enables tumor immune escape. A study has indicated that inhibiting the A2A receptor may enhance the anti‐tumor efficacy of CAR‐T cells, highlighting its prospective role as a target in the design of CAR‐T cell therapies.^[^ ^60^ ^]^ The A2A receptors interact with adenosine, leading to the suppression of immune cell activity and facilitating tumor evasion from immune responses. Inhibition of the A2A receptor appears to bolster the anti‐tumor capabilities of CAR‐T cells, suggesting that targeting A2A may be a viable strategy for enhancement of CAR‐T cells.^[^ ^6^ ^]^ By utilizing the functional properties of GPCRs, it becomes feasible to engineer modified constructs that aim to elevate the diverse functionalities of CAR‐T cells. Such strategic alterations show the potential to remarkedly enhance their effectiveness or enable them to overcome the immunosuppressive milieu within the TME. GPR65 exhibits dual roles in antitumor immunity. The elevated expression of GPR65 may enhance T cell‐mediated tumor killing by suppressing protumor factors and sustaining proinflammatory M1 phenotype in immune cells (e.g., macrophages). Whereas GPR65 deficiency may expand immunosuppressive M2 macrophages in the TME and impair T cell function via immunosuppressive factor secretion, thereby promoting immune evasion. Adaptive immunotherapies demonstrate high sensitivity to singular tumor‐expressed genes through tumor‐TME interactions and TME reprogramming. GPR65 expression may serve as a biomarker to predict CD19 CAR‐T cell therapy response in B‐ALL patients. Combination of CAR‐T therapy and anti‐VEGFA treatment represents a potential strategy to overcome drug resistance. However, direct genetic editing of CAR‐T cells to modulate GPR65 requires careful consideration.^[^ ^61^ ^]^ In HNSCC, the expression of S1PR4 positively correlates with the proportion and function of CD8+ T cells, indicating its importance in regulating the TME.^[^ ^62^ ^]^ Consequently, the development of S1PR4‐targeted CAR‐T cells may facilitate improved T cell localization to tumors, thereby enhancing therapeutic outcomes.

### 7.4 The GPCR‐Targeted SynNotch Gating System and GPCR as Sensor and Switch

While demonstrating remarkable efficacy for the intervention of mutiple diseases, CAR‐T cell therapy also faces increasing safety concerns arising from three primary aspects. First, as “living drugs,” their long‐term persistence in vivo may lead to unpredictable delayed effects. Second, excessive activation can trigger acute clinical toxicities such as CRS and ICANS. Third, on‐target/off‐tumor toxicity may inadvertently damage normal tissues expressing the target antigen. To precisely control the spatiotemporal activity of CAR‐T cells and promptly terminate toxic responses, researchers have developed various controllable “switch” strategies. To address the safety challenge, researchers have developed various small‐molecule‐based regulatory strategies. Among these, ON‐switch systems achieve reversible drug‐dependent activation by inducing dimerization of split CAR components, though functional maintenance requires continuous drug presence; OFF‐switch systems rapidly suspend activity by disrupting CAR assembly or signaling, but impose stringent requirements on drug pharmacokinetics; suicide gene systems achieve complete CAR‐T cell elimination by inducing caspase activation, providing an ultimate safety barrier, though their action is irreversible and potentially susceptible to escape. Combinatorial implementation of these control systems may enable precise management of therapeutic safety while preserving the durable efficacy of CAR‐T cells.^[^ ^63^ ^]^

SynNotch receptors can also be employed to control CAR‐T cell expression, with a design inspired by the core architecture and activation mechanism of the native Notch signaling pathway. The native Notch receptor is a single‐pass transmembrane protein whose extracellular domain undergoes sequential cleavage by ADAM metalloprotease and γ‐secretase upon ligand binding, resulting in the release of its intracellular domain into the nucleus to regulate downstream gene transcription. SynNotch receptors undergo modular reconstruction based on this framework: first, their extracellular recognition domain is replaced with programmable antigen‐binding modules such as single‐chain variable fragments (scFv), nanobodies, or designed ankyrin repeat proteins, enabling specific recognition of user‐defined cell surface antigen A; second, their intracellular transcriptional effector domain is replaced with artificial transcription factors (e.g., Gal4‐VP64), enabling activation‐induced expression of user‐defined transgenes. Based on this core principle, researchers have constructed various logic‐gated systems (AND, OR, and NOT gates) to achieve precise spatiotemporal control of T cell cytotoxic activity (Figure 3D).^[^ ^64^ ^]^ For example, a SynNotch‐based dual‐target system recognizes the vascular endothelial marker Apj (a GPCR family member) and induces CAR‐T expression specifically in the tumor vasculature microenvironment, enabling precise spatially dependent killing.^[^ ^65^ ^]^

Building upon the functional recruitment of GPCRs by synthetic biology systems like SynNotch, researchers have further consider the direct engineering of GPCRs to enables their utilization as programmable cellular sensors. This concept faces a fundamental challenge: GPCR structures inherently lack modularity, and their ligand specificity engineering has long relied on time‐consuming and complex structural mutagenesis and molecular evolution. To overcome this limitation, Ting et al. innovatively integrated nanobodies with conditional autoinhibition domains, developing a flexible, modular strategy for precise receptor regulation (Programmable antigen‐gated G‐protein‐coupled engineered receptors, PAGER) (Figure 3D). The core design involves a introduction a conditional autoinhibition module composed of nanobodies into the extracellular region of GPCRs, maintaining the receptor in a quiescent state in the absence of specific exogenous antigens, whereas upon target antigen binding to the nanobody, this autoinhibition is specifically relieved, thereby precisely triggering downstream signaling. This reconfiguration not only prevents nonspecific receptor activation but also achieves externally programmable control over its activity without requiring complex structural modifications to the GPCR, opening new synthetic biology pathways for GPCR functional studies and therapeutic applications.^[^ ^66^ ^]^

## 8 Future Directions of GPCR CAR‐T Therapy

### 8.1 Targeting GPCR Complexes

**Figure 4:**
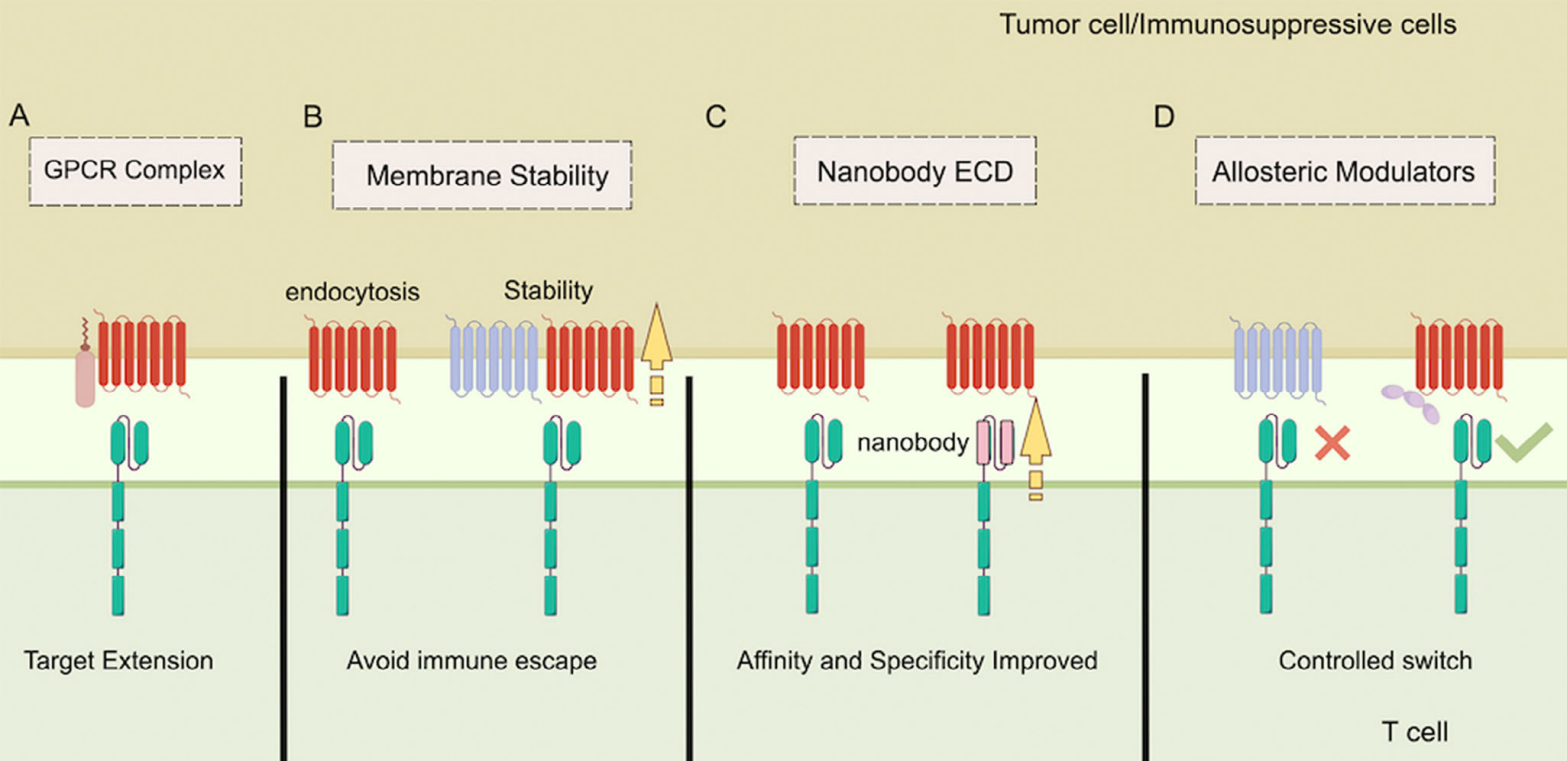
Optimization strategies for future GPCR CAR‐T cells. A) Targeting complexes: CAR‐T cells target GPCR complexes, improving specificity and efficacy by recognizing multiple epitopes. B) Improve membrane expression: Accessory proteins, such as LPAR1 modulated to enhance the surface expression of GPRC5D on tumor cells. C) AI can further optimize the affinity and specificity of the extracellular domain of nanobodies in CAR‐T cells. D) Allosteric modulators are used to induce specific conformational states of GPCRs, enhancing the binding affinity and specificity of CAR‐T cells and improving the safety of the therapy. By Figdraw: APSII09009.

GPCR receptor complexes consist of GPCRs and their interacting proteins, which not only regulate the conformation and function of the receptors but also influence their subcellular localization and signaling specificity (**Figure** **4**A). Erenumab, a monoclonal antibody approved by the FDA for migraine treatment, targets the complex formed by the calcitonin receptor‐like receptor (CLR) and receptor activity‐modifying protein 1 (RAMP1).^[^ ^67^ ^]^ The unique conformational and functional diversity of GPCR complexes provides new targets for drug development. Insight into the unique subcellular localization of GPCR complexes could help CAR‐T cells to recognize tumor cells with more precise specificty, thereby reducing off‐target effects. Despite technical challenges such as complex stability, antigen screening, and in vivo delivery, the rapid development of cryo‐electron microscopy, artificial intelligence, and synthetic biology have revealed significant clinical translational potential in this field. In the future study, CAR‐T cells targeting GPCR complexes are expected to become an important development direction for tumor immunotherapy.

### 8.2 Targeting GPCR Dimerization Networks to Block CAR‐T Immune Escape

The phenomenon of tumor immune evasion poses a significant obstacle to CAR‐T cell therapy. The primary mechanism underlying this challenge involves the downregulation or complete loss of expression of target antigens. This alteration hinders the ability of CAR‐T cells to identify and subsequently eradicate tumor cells (Figure 4B). Classical models assume GPCRs function as monomers, but GPCRs also exist as homodimers or heterodimers, and multimerization can alter GPCR ligand binding, second messenger activation, and cell surface trafficking.^[^ ^68^ ^]^ For instance, the enhanced expression of the δ‐µ opioid receptor on the cell surface is modulated by GPCR complexes.^[^ ^69^ ^]^ Additionally, arrestin pathway‐selective angiotensin II type 1 receptor (AT1) agonists promote the internalization of AT1‐B2 receptor heterodimers, modulating B2 bradykinin receptor signaling.^[^ ^70^ ^]^ Our previous research on GPCR dimers revealed that the scaffold protein MARP2 interacts with 46 GPCRs and directly regulates the membrane localization of some GPCRs through dimerization.^[^ ^71^ ^]^ Furthermore, metformin upregulates GPRC5D expression in MM cells, improving the therapeutic effects of GPRC5D‐CAR‐T cells. Further research found that lysophosphatidic acid receptor 1 (LPAR1) can form heterocomplexes with GPRC5D. When the LPAR receptor is activated by its natural agonist 1‐Oleoyl‐LPA, it induces GPRC5D internalization and significantly reduces its membrane surface expression, thereby impairing the antigen recognition ability of CAR T cells.^[^ ^72^ ^]^ Further experiments confirmed that combining LPAR‐specific antagonists effectively blocks this process, reverses GPRC5D internalization, and significantly increases its membrane localization, ultimately restoring the killing efficacy of CAR T cells against MM. Therefore, based on the mechanism of GPCR heterodimerization regulating membrane antigen expression homeostasis, further exploration of the membrane stabilityregulatory networks of numerous GPCRs may overcome antigen escape challenges in CAR‐T therapy by maintaining membrane antigen expression homeostasis.

### 8.3 Binding of nanobody to Extracellular Domains Enhance GPCR CAR‐T Safety and Efficacy

Nanobody (Nbs), also known as VHH, are single‐domain antibodies composed of the variable region of heavy‐chain‐only antibodies found in camelids, with a size only one‐tenth that of conventional antibodies. Unlike traditional antibodies, Nbs lack light chains but retain strong binding capabilities. The structure of Nbs adopts a β‐sandwich fold, supporting three complementarity‐determining regions (CDRs), which are critical for antigen binding. Due to the convex shape of nanobody paratopes and their flat structure, Nbs can recognize and bind to antigenic sites that are inaccessible to traditional antibodies.^[^ ^24^ ^]^ Additionally, Nbs offer advantages such as small size, structural stability, strong binding affinity, ease of production, and low immunogenicity, making them highly promising for biomedical applications.^[^ ^73^ ^]^ Studies have shown that Nbs exhibit better stability and stronger antigen recognition capabilities compared to scFv (Figure 4C). The absence of a linker peptide in Nbs allows them to avoid CAR surface aggregation and non‐antigen‐dependent toxicity more effectively, making them more suitable for the extracellular domain of CARs.^[^ ^74^ ^]^ Clinical trials of results for the GPRC5D CAR‐T therapies MCARH109 and OriCAR‐017 have been released recently: MCARH109 follows the classic scFv design, while OriCAR‐017 adopts a VHH‐VHH structure. Notably, compared to the traditional scFv structure, the VHH‐VHH structure demonstrates significant advantages in key metrics such as safety and efficacy.^[^ ^15^, ^19^ ^]^ Thus, engineering nanobody‐based targeting domains is crucial for CAR‐T to break through the therapeutic efficacy barriers of existing treatments and enhance safety.

### 8.4 Allosteric Modulators of GPCRs as CAR‐T Switches to Enhance Safety

In solid tumors, the recognition of non‐tumor tissues expressing target antigens by CAR‐T cells often leads to clinically significant off‐tumor toxicity, referred to as OTOT. Various strategies have been developed to limit the off‐tumor toxicity of CAR‐T cells. One strategy involves expressing an inducible caspase‐9 suicide gene (icaspase9) in CAR‐T cells, which can be activated by the small molecule drug AP1903 to induce apoptosis in CAR‐T cells.^[^ ^75^ ^]^ Alternatively, additional cell surface molecules that can be recognized by antibodies can be expressed on CAR‐T cells, allowing the utilization of monoclonal antibodies to target and eliminate CAR‐T cells, leading to irreversible elimination of CAR‐T cells. Another option is to design a reversible on/off switch that allows for temporal control of CAR‐T cell activation while preserving anti‐tumor functionality. By fusing a protease recognition site, protease, and degradation factor to the C segment of the CAR, the protease can cleave itself and the degradation factor from the CAR in the absence of the protease inhibitor Asunaprevir, preventing CAR degradation and maintaining its expression on the cell surface. The presence of Asunaprevir inhibits the protease cleavage, thereby leading to the shutdown of CAR functionality. Additionally, a common strategy is to establish logic‐gated CAR‐T cells that utilize switches like “IF/THEN,” “AND,” “OR,” and “NOT” to flexibly control CAR‐T cell activation.^[^ ^76^ ^]^ However, all of these strategies require additional editing of CAR‐T cells, complicating the manufacturing process and potentially impacting CAR‐T cell functionality. Allosteric modulators usually bind to non‐conserved regions such as the transmembrane domain or lipid membrane interface of receptors, causing conformational changes or alterations in protein dynamics to achieve subtype‐specific regulation.^[^ ^77^ ^]^ Allosteric modulators of GPCRs possess unique pharmacological advantages, reflected in their ability to maintain the signaling properties of endogenous ligands, reduce side effects, broaden the signaling range, and even restore the function of pathogenic mutant receptors.^[^ ^21^ ^]^

This review paper suggests that allosteric modulators of GPCRs may be employed to mitigate OTOT. Theoretically, when off‐target toxicity occurs, the addition of GPCR allosteric modulators can induce specific conformational changes in GPCRs, preventing CAR‐T cells from binding to their targets (Figure 4D). This regulatory mechanism not only allows for controllability and reversibility in the treatment process but also significantly enhances the safety of CAR‐T cell therapy and the flexibility of its clinical application, providing new strategies and ideas for tumor immunotherapy. Currently, this strategy has not been studied, primarily due to significant bottlenecks in the development of GPCR allosteric modulators, including a lack of systematic screening and pharmacological characterization, as well as a limited understanding of the mechanisms of allosteric modulation. Trends in GPCR drug development indicate that new drugs are increasingly focused on allosteric modulators compared to all approved orthosteric medications. Recently, researchers discovered an allosteric nanobody for the adhesion GPCR ADGRG2 and proposed a screening strategy for allosteric Nbs targeting adhesion GPCRs, revealing their allosteric regulatory mechanisms and providing a template for developing Nbs as allosteric modulators. The study employed nanobody yeast display system and obtained the nanobody Nb23 that could specifically binds to the ADGRG2‐NTF structural domain through magnetic bead selection and flow sorting. Furthermore, by fusing the nanobody Nb23, a stronger affinity nanobody Nb23‐bi was generated, with a Kd of 0.46 ± 0.05 µM for binding to ADGRG2.^[^ ^78^ ^]^ Moreover, the researchers found that Nb23‐bi can synergistically enhance the efficacy of DHEA by stimulating the downstream Gs and Gq signaling pathways induced by ADGRG2. Based on the discovery of allosteric sites in GPCR, as well as the discovery and design of ligand – or structure‐based allosteric drugs, several related drugs have been approved for marketing, including Avacopan, Cinacalcet, and others.^[^ ^79^ ^]^ Additionally, two notable GPCR allosteric modulators have entered clinical stages: emraclidine, which targets the M4 receptor for the treatment of schizophrenia, and SBI‐553, which targets NTSR1 to alleviate addiction issues. Currently, numerous additional compounds are currently undergoing evaluation in phase I to III clinical trials. The above studies provide possibilities for the future development of this strategy. Finally, the application of allosteric modulators needs to focus on their selectivity and safety to avoid interference with normal cell functions. Additionally, their stability in vivo and pharmacokinetic properties need to be considered to ensure effective therapeutic outcomes.

## 9 GPCR Immunotherapy: Beyond CAR‐T Cells

The rapid advancement of diverse GPCR‐targeting therapies beyond CAR‐T cells provides critical target validation and valuable engineering insights for the development of GPCR‐directed CAR‐T strategies. These modalities demonstrate the clinical tractability of GPCRs as tumor antigens and offer potential avenues for combination therapies.

### 9.1 Antibody‐Derived Therapeutics

The success of monoclonal antibodies, bispecific antibodies, antibody‐radionuclide conjugates (ARCs), and antibody‐drug conjugates (ADCs) targeting GPCRs directly validates these receptors as viable tumor‐associated antigens. For instance, the bispecific antibody talquetamab (targeting GPRC5D and CD3) and the ADC AZD0305 (targeting GPRC5D) have shown promising efficacy in multiple myeloma, confirming GPRC5D's therapeutic relevance. Furthermore, the approval of peptide receptor radionuclide therapy (PRRT) targeting somatostatin receptors (177Lu‐DOTATATE) for neuroendocrine tumors underscores the potential for precise tumor targeting via GPCRs. The scFvs derived from these validated antibodies serve as ideal candidates targeting domains for the extracellular moiety of GPCR‐directed CARs. Representative GPCR antibody‐based therapeutics that have advanced to Phase II/III clinical studies are summarized in **Table** **4**.

**Table 4:** Antibody‐related drugs targeting GPCRs that have entered Phase II and III clinical trials or have been approved.

Type	Agent	Target	Indications	Phase	CTID	Refs.
Monoclonal antibody	Avdoralimab	C5aR1	Bullous Pemphigoid	II	NCT04563923	[163]
	Erenumab	CGRP	Migraine	Approved	BLA 761077	[164]
	Glutazumab	GLP‐1R	obesity and type 2 diabetes	III	CTR20222558	[165]
	Leronlimab	CCR5	TNBC	II	NCT03838367	[166]
	Mogamulizumab	CCR4	T‐cell leukemia‐lymphoma	Approved	BLA 761051	[167]
	Nimacimab	CB1R	diabetic gastroparesis	IIa	NCT03900325	[168]
	Nimacimab	CB1R	Obesity	IIa	NCT06577090	[169]
	Plozalizumab	CCR2	diabetic nephropathy	II	NCT02410499	[170]
	Plozalizumab	CCR2	Atherosclerotic cardiovascular disease	II	NCT00715169	[171]
	Ulocuplumab	CXCR4	Relapsed Multiple Myeloma(	II	NCT01359657	[172]
	Volagidemab	GCGR	T1D	II	NCT03117998	[173]
Bispecific antibody	Petosemtamab	EGFR LGR5	Head and Neck Squamous Cell Carcinoma	III	NCT06496178	[174]
	Talquetamab	CD3 GPRC5D	Relapsed or Refractory Multiple Myeloma	III	NCT05455320	[175]
	Tidutamab	CD3 SSTR2	Advanced Merkel Cell Carcinoma or Extensive‐stage Small Cell Lung Cancer	I/II	NCT04590781	[176]
ADC	TAK‐500	CCR2	select locally advanced or metastatic solid tumors	I/II	NCT05070247	[177]

### 9.2 Small Molecule Drugs & Novel Platforms

Small molecule agonists or antagonists can modulate the tumor immune microenvironment by targeting GPCRs involved in immune cell migration and function. More innovatively, these small‐molecule ligands can be engineered into nanobody formats for secretion by CAR‐T cells, enabling precise, localized modulation of specific GPCR pathways in the tumor niche and significantly expanding CAR‐T functional scope. Similarly, oncolytic viruses engineered to express GPCR‐targeting nanobodies (e.g., a CXCR4‐targeted oHSV)^[^ ^80^ ^]^ and cancer vaccines targeting GPCRs (e.g., an FSHR DNA vaccine)^[^ ^81^ ^]^ have demonstrated the ability to remodel the TME and enhance T‐cell infiltration. These approaches highlight promising combination strategies where GPCR‐CAR‐T cells could be partnered with these modalities to overcome challenges in solid tumors, such as immunosuppression and inadequate T‐cell trafficking.

## 10 Conclusion and Outlook

**Figure 5:**
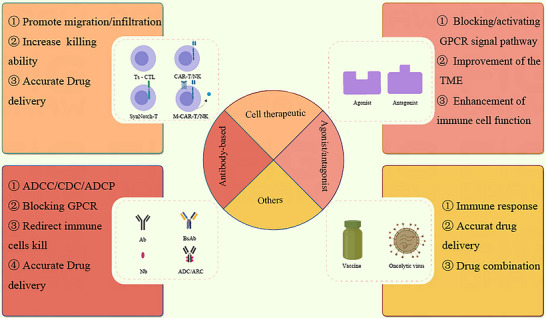
Cancer immunotherapy and related mechanisms targeting GPCR. GPCR immunotherapy mainly includes cell therapy, antibody therapy, antagonists/agonists, and others; they can also be combined with other therapies like Immune checkpoint inhibitors. Abbreviations: CTL, cytotoxic lymphocyte; CAR, Chimeric Antigen Receptor; mAb, monoclonal antibody; BsAb, Bispecific Antibodies; ADC, antibody‐drug conjugate.

CAR‐T cell therapy is primarily employed to treat various cancers, particularly hematologic malignancies, where it has demonstrated remarkable effectiveness. However, CAR‐T therapy targets are highly homogeneous, with approved drugs targeting only two targets, CD19 and BCMA. Prolonged single‐target therapy can lead to immune escape and drug resistance. In addition, the indications of CAR‐T therapy are greatly limited due to the single target. In recent years, with the deepening of the understanding of the structure of GPCRs and the development of antibody preparation technology, GPCRs have attracted more and more attention as novel targets for CAR‐T cell therapy. At present, GPRC5D CAR‐T cells have shown significant efficacy in a few clinical trials for relapsed and refractory MM, which provide a strong confidence for the future development of GPCR CAR‐T cells. In recent years, the rapid development of single‐cell sequencing and high‐throughput screening technologies has provided more powerful approaches for systematic discovery of GPCR targets. In the future, we are expected to develop a comprehensive dynamic expression profile of GPCRs in the TME. By constructing a network database of GPCR expression across tumor types, researchers can accurately identify specific targets with therapeutic potential. Establishment of a standardized GPCR antibody drug development system is crucial for the development of GPCR CAR‐T therapy. The cost of drug development determines the accessibility of the drug for clinical application. Notably, some enterprises are striving to establish a standardized development process for GPCR antibody drugs, and GPCR CAR‐T therapy is also expected to achieve the construction of a full‐chain development platform in the future. In addition, ingenious utilization of the intrinsic signal transduction characteristics of GPCRs provides new designs for optimizing the function of CAR‐T cells. By regulating the signaling pathways mediated by GPCRs, the activation, proliferation, and persistence of CAR‐T cells can be enhanced, while improving TME. It provides more possibilities to solve the bottleneck of CAR‐T cell therapy. It is worth noting that the high heterogeneity of GPCRs families and the structural similarity of the same subfamily pose severe challenges for clinical application. The differential expression of receptor subtypes in different tumor types, the dynamic changes of the microenvironment, and the bidirectional regulation of some GPCRs in the activation and exhaustion of T cells require that therapeutic strategies must be carefully evaluated according to the specific disease types and clinical test results. Although there are many scientific difficulties, the strategy of targeting GPCRs has provided a promising way to break through the current treatment bottleneck. Compared to traditional small‐molecule drugs, immunotherapy mobilizes the body's immune system to recognize and eliminate tumor cells, offering greater specificity and longer‐lasting anti‐tumor effects, minimizing damage to normal cells while overcoming resistance seen in conventional therapies. Besides CAR‐T cell therapy, this review also explores various GPCR‐based immunotherapy strategies, including CAR‐NK, antibody therapy, GPCR agonists and antagonists, as well as vaccines and oncolytic viruses. Each of these methods has its unique characteristics and achieves tumor cell elimination through different mechanisms (Figure 5). For instance, cell therapy can recognize and bind to GPCR targets, directly attacking and destroying cancer cells or immunosuppressive cells that express these targets; antibody therapy works by marking or blocking GPCR signaling pathways, thereby inhibiting tumor growth or modulating the TME; agonists and antagonists adjust GPCR signaling, influencing the activity of immune cells or the survival environment of tumor cells. The coexistence and development of various immunotherapy methods stem from the heterogeneity and complexity of cancer—single therapies often struggle to effectively address all patients and tumor types. The coexistence and advancement of different therapies promote the development of personalized treatment, allowing a cocktail therapy to be optimized based on individual patient conditions, thereby enhancing efficacy and reducing side effects. Various immunotherapy strategies drive scientific progress, enabling more therapies to enter clinical practice and providing patients with a wider range of options. Many studies on GPCR‐based therapies have yielded encouraging results; however, numerous GPCR targets remain unexplored, indicating substantial untapped potential—these undeveloped targets may lead to new breakthroughs and innovations in future immunotherapy, further enhancing the effectiveness and safety of cancer treatments. Looking forward, GPCR CAR‐T therapy combined with other immunotherapy is expected to play a greater role in cancer treatment. As research continues to deepen, more GPCR targets are expected to be discovered and utilized, leading to more diverse and precise treatment options. These advancements are poised to contribute to an increased success rate of cancer therapies and establish a foundation for breakthroughs in GPCR‐CAR‐T therapy for solid tumors and other autoimmune diseases, thereby offering more therapeutic options to improve patient outcomes clinical interventions.

## Author Contributions

Z.L., Y.X., Y.Z., and L.D., contributed equally to the work. C.Z., L.C., F.T., and B.L. designed the study. Z.L. served as the primary author, responsible for creating the figures and preparing the tables. Y.X., Y.Z., and L.D. drafted the manuscript and conducted revisions. J.W.H.W., D.J.H.S., S.L., H.T., and L.L. provided valuable support and insights. All authors reviewed and approved the final manuscript.

## Conflict of Interest

The authors declare no conflict of interest.
